# School environment as predictor of teacher sick leave: data-linked prospective cohort study

**DOI:** 10.1186/1471-2458-12-770

**Published:** 2012-09-11

**Authors:** Jenni Ervasti, Mika Kivimäki, Ichiro Kawachi, SV Subramanian, Jaana Pentti, Tuula Oksanen, Riikka Puusniekka, Tiina Pohjonen, Jussi Vahtera, Marianna Virtanen

**Affiliations:** 1 Finnish Institute of Occupational Health, Centre of Expertise for Work Organizations, Topeliuksenkatu 41 a A, 00250, Helsinki, Finland; 2 Department of Epidemiology and Public Health, University College London, 1-19 Torrington Place, London, WC1E 6BT, UK; 3 Department of Behavioral Sciences, PB 9, 00014 University of Helsinki, Helsinki, Finland; 4 Harvard School of Public Health, Department of Society, Human Development and Health, 677 Huntington Avenue, Kresge Building 7th Floor, 716 Boston, Massachusetts, 2115-6096, USA; 5 National Institute for Health and Welfare, PB 30, 00271, Helsinki, Finland; 6 City of Helsinki, Occupational Health Centre, PB 5603, 00099, Helsinki, Finland; 7 Department of Public Health, University of Turku, Lemminkäisenkatu 1, 20014, Turun yliopisto, Finland; 8 Turku University Hospital, PB52, 20521, Turku, Finland

**Keywords:** Bullying, Multilevel, Perceived indoor air, School satisfaction, Ventilation

## Abstract

**Background:**

Poor indoor air quality (IAQ) and psychosocial problems are common in schools worldwide, yet longitudinal research on the issue is scarce. We examined whether the level of or a change in pupil-reported school environment (IAQ, school satisfaction, and bullying) predicts recorded sick leaves among teachers.

**Methods:**

Changes in the school environment were assessed using pupil surveys at two time points (2001/02 and 2004/05) in 92 secondary schools in Finland. Variables indicating change were based on median values at baseline. We linked these data to individual-level records of teachers’ (n = 1678) sick leaves in 2001–02 and in 2004–05.

**Results:**

Multilevel multinomial logistic regression models adjusted for baseline sick leave and covariates showed a decreased risk for short-term (one to three days) sick leaves among teachers working in schools with good perceived IAQ at both times (OR = 0.6, 95% CI: 0.5-0.9), and for those with a positive change in IAQ (OR = 0.6, 95% CI: 0.4-0.9), compared to teachers in schools where IAQ was constantly poor. Negative changes in pupil school satisfaction (OR = 1.8, 95% CI: 1.1-2.8) and bullying (OR = 1.5, 95% CI: 1.0-2.3) increased the risk for short-term leaves among teachers when compared to teachers in schools where the level of satisfaction and bullying had remained stable. School environment factors were not associated with long-term sick leaves.

**Conclusions:**

Good and improved IAQ are associated with decreased teacher absenteeism. While pupil-related psychosocial factors also contribute to sick leaves, no effect modification or mediation of psychosocial factors on the association between IAQ and sick leave was observed.

## Background

Health problems related to poor indoor air quality (IAQ), sometimes referred to as sick-building syndrome or building-related symptoms, include headache, nausea, eye, nose and throat problems, chest tightness or shortness of breath, fatigue, chills and fever, dizziness, dry skin, or even clusters of serious health problems [1]. In Finnish office worker population (n = 11 154), the most common symptoms of poor indoor air were irritated, stuffy, or runny nose (20%), itching, burning, or irritation of the eyes (17%), and fatigue (16%) [2]. These symptoms are often relatively minor, non-specific, and common amongst the general population. Despite their minor nature, these symptoms may have a great effect on public health and incur costs to the economy through widespread absenteeism and lowered productivity among the affected workers [3,4].

Possible sources of indoor air pollutants are building materials, furniture, office equipment, heating, ventilation, air conditioning, detergents used for cleaning, and pollutants originating from people and their activities [5]. Increasing outdoor ventilation, removing or managing sources of contaminants, and increasing air cleaning or filtration have shown beneficial health effects [6].

The quality of IAQ and ventilation are often assessed using objective measures such as CO_2_ or NO_2_ concentrations [3,7-9]. Although these measures are reliable and valid, they measure the IAQ from a narrow point of view and large-scale studies are expensive and time-consuming. Subjective reports of IAQ, i.e., perceived IAQ, provide a wider scope of potential problems and subjective experience of symptoms. Good perceived IAQ has been associated with cool and dry air, [10] low pollution loads in the office [11], good ventilation systems [12-14], the use of air purifiers [5], and low levels of volatile organic compounds (VOCs) [15].

However, with regard to the health effects of poor perceived IAQ, IAQ reports and the measured outcomes (health symptoms) usually come from the same source, leaving the results open to common-method bias. Perceived IAQ had only weak effects on health at follow-up in prospective, cross-lagged design. The results however indicated a reversed effect of health problems leading to increased IAQ complaints, which led the authors to conclude that having pre-existing health problems may lead to increased IAQ complaints even when adjusting for the overall level of complaining [16].

It has been proposed that psychosocial stress, i.e. pathophysiological changes related to psychological responses to the social environment [17], possibly mediates environmental symptoms by altering the body’s sensitivity to physical demands and toxic exposures. Research has linked psychosocial factors quite strongly with building-related symptoms, especially among women [18,19], but not on psychosocial factors alone: most of the studies have supported the conclusion that these symptoms are of multifactorial origin related to chemical, physical, biological and psychosocial factors [18-23].

IAQ problems, such as poor ventilation, are common in schools worldwide [6,24-26]. Also pupil-related psychosocial problems are rather common, and they have shown to negatively associate with teachers’ health and subsequent sick leave [27,28]. Low pupil cohort socioeconomic composition at school [29] and lack of school resources as measured by pupil-teacher ratio (PTR) [30] have also been associated with teacher sick leave. Moreover, school location, i.e., school urbanicity, low pupil socioeconomic composition, and high PTR have been associated with poor IAQ [26].

In this study, we used pupil evaluations of IAQ in school as a predictor of the subsequent health problems of their teachers, as indicated by sick leave. Specifically, we aimed to test whether the level of or a change in pupil-evaluated IAQ was predictive of teacher sick leave when prior sick leaves and changes in the pupil-related psychosocial factors, pupil school satisfaction [27] and bullying [28], were controlled for. Furthermore, we controlled for changes in pupil cohort socioeconomic composition and in the PTR at school.

## Methods

### Sample and procedure

We linked data from two independent data sources in Finland: (1) the School Health Promotion Study (SHPS), which is a classroom survey of eighth and ninth grade pupils from lower secondary schools aged 14 to 16, and (2) employer registers of the same schools on teachers’ sex, age, job contract, occupation, and sick leaves, i.e. data extraction conducted in the Finnish Public Sector Study (FPSS). The SHPS was approved by the Ethics Committee of the Pirkanmaa Hospital District and the FPSS was approved by the Ethics Committees of Helsinki and Uudenmaa Hospital District and the Finnish Institute of Occupational Health.The data have been described elsewhere in detail [27-30].

The sample of the present study consisted of 1758 lower secondary school teachers with register data on sick leaves, who had been working in the same schools both in school year 2001–02 and 2004–05. We excluded 80 teachers with missing information on the SHPS. Thus, the final analytic sample consisted of 1678 teachers working in 93 lower secondary schools in nine municipalities in Finland (95% of the original teacher sample).

### Measures

#### Perceived IAQ

Pupil perceptions of IAQ were measured through a questionnaire survey at the following time points: the first measurement was conducted in April 2001 (27 schools in five towns) or in April 2002 (65 schools in three towns). The second measurement was carried out in April 2004 (65 schools in 3 towns) or in April 2005 (27 schools in 5 towns). The number of pupils participating in these measurements was above 20 000 on both times. Perceived IAQ was measured using one question in a list of various physiological and psychological exposures at school: “Do any of the following factors hinder your school work? Poor ventilation or indoor air”. The scale was from 1 to 4, in which 1 = not at all, 4 = very much. These measures were aggregated to school-level means of IAQ. Change in the perceived IAQ variable was calculated using the median split at baseline (in 2001/02). The median of perceived IAQ in 2001/02 was 2.72. The following categories were then formulated: 1 = Poor IAQ at both times (above median at both time points, reference group); 2 = Good IAQ at both times (below median at both time points); 3 = From good to poor IAQ, i.e., negative change (below median in 2001/02 and above median in 2004/05); 4 = From poor to good IAQ, i.e., positive change (above median in 2001/02 and below median in 2004/05).

#### Teacher sick leaves

We obtained individual-level data on teacher sick leave during school years (from September to May) 2001–02 and 2004–05 from employers’ registers. Using these records, we calculated the number of short-term (one to three days, self-certified) and long-term sick leave (over three days, medically certified) spells for each teacher. The multinomial variable (type of sick leaves in 2004–05) was as follows: 0 = no sick leaves (reference group); 1 = short-term absences only; 2 = long-term absences (with or without short-term sick leaves).

#### Covariates

Individual-level covariates included teachers’ age, sex, type of job contract (permanent/fixed term), occupation (general education teacher/special education teacher/headmaster) at follow-up (2004–05), and teacher sick leave types at baseline (2001–02). School-level covariates included PTR (=number of pupils at school/number of teachers at school); pupil cohort socioeconomic composition (the percentage of pupils at school whose mother had no higher than a vocational education); pupils’ school satisfaction (Do you like going to school? Scale of 1–4, in which 1 = Very much, 4 = Not at all [school-level mean]); and bullying at school, two questions (percentage of pupils being bullied at least once a week and percentage of pupils that have bullied at least once a week). For analysis, these measures were coded as binary variables using the median split at baseline.

The level of or change in these variables describing PTR, pupil cohort socioeconomic background, and school psychosocial environment were calculated in a similar way to the perceived IAQ variable. The medians at baseline were: pupil-teacher ratio = 10.55; pupil cohort socioeconomic composition = 44.70; school satisfaction = 2.49; pupils being bullied = 7.14; pupils bullying others = 5.95.

Pupil socioeconomic composition was higher in 2001/02 than in 2004/05 in only two schools (with 41 teachers). We therefore combined these teachers with teachers in schools in which pupil socioeconomic composition was lower than median at both times (27%, n = 453). Teachers in schools in which pupil socioeconomic composition was higher than median at both times (48%, n = 807) formed the reference group. In addition, 418 teachers (25%) were in schools with a change from lower to higher pupil socioeconomic composition.

There were 175 teachers in schools with below median PTR at both times, and 48 teachers in schools with a positive change in PTR. These teachers, totaling 13% of the study group formed the reference group. A total of 47% (n = 786) teachers worked in schools where the PTR was above the median at both times, and 40% (n = 669) worked in schools where the PTR had increased (i.e., negative change).

Since the follow-up time was 2 years for the schools in Helsinki metropolitan area (3 towns, 65 schools), and 4 years for the rest of the schools, we controlled also for the school location, i.e., the follow-up time (1 = 4 years, 2 = 2 years).

### Statistical analyses

We analyzed the change in perceived IAQ during the follow-up using frequencies, means and a paired samples *t*-test. Because individual teachers were nested in schools and schools in municipalities, we used a multilevel data structure with teachers at the 1st level, schools at the 2nd level, and municipalities at the 3rd level (SAS software, GLIMMIX Procedure). We used multinomial logistic regression models to examine the risk of sick leave and to estimate the odds ratios (OR) with their 95% confidence intervals (CI) for variables of change in perceived IAQ, adjusting for individual- and school-level covariates. We also estimated the variance components (random effects) of type of sick leave in order to take into account the school- and municipality-level variance.

## Results

Table 1 presents the descriptive statistics. The PTR was high at both times in 77% of schools where a negative change in IAQ had occurred. However, also the pupil cohort socioeconomic composition was high in 67% of those schools. Schools in which the perceived IAQ was good at both times had fewer negative changes in pupil school satisfaction. Negative changes IAQ seemed to somewhat co-occur with negative changes in bullying.

**Table 1:** Descriptive statistics of study sample by the level of or change in indoor air quality (IAQ) as evaluated by pupils

**2004/05 Teacher characteristics**	**All, 93 schools (n = 1678)**	**1 Poor IAQ at both times, 28 schools (n = 485)**	**2 Good IAQ at both times, 37 schools (n = 699)**	**3 Negative change; from good to poor AIQ, 7 schools (n = 121)**	**4 Positive change from poor to good IAQ, 21 schools (n = 373)**	P for difference between groups*****
Mean age, years	47.9	47.6	48.1	48.4	47.6	0.57
Percentage of male teachers	26.5	25.0	29.0	21.5	25.5	0.20
Percentage of teachers with fixed-term job contracts	14.0	14.6	13.6	20.7	11.8	0.10
Percentage of special education teachers	5.5	6.2	5.7	1.7	5.6	0.58
**Percentage of teachers in schools with**						
high PTR*	46.8	50.5	36.3	76.9	52.0	<0.001
high pupil cohort socioeconomic composition**	48.1	36.7	47.6	66.9	57.6	<0.001
Negative change***						
school satisfaction	13.8	20.8	4.3	14.1	22.5	<0.001
pupils being bullied	20.9	28.0	20.6	25.6	10.5	<0.001
pupils bullying others	17.1	16.5	15.2	36.4	15.3	<0.001
School location/follow-up time****						
Metropolitan area	66.5	77.9	53.7	100	64.9	<0.001
Other	33.5	22.1	46.3	0	35.1	

* Indicates above baseline median pupil-teacher ratio both at baseline and at follow-up.

** Indicates the percentage of pupils at school whose mothers had higher than a vocational education both at baseline and at follow-up (high pupil cohort socioeconomic composition).

*** Indicates a change from baseline (2001/2002) median to follow-up (2004/2005).

**** Helsinki metropolitan area: 2-year follow-up; Other: 4-year follow-up.

***** *x*^2^ test for nominal variables, ANOVA for continuous variable (least square means).

The school location (metropolitan area vs. other) indicated also the follow-up time. In the schools located in the Helsinki metropolitan area (with a 2 year follow-up), the perceived IAQ was more often poor at follow-up than in schools located in other towns (with a 4 year follow-up). The negative changes in the IAQ all occurred in schools located in the Helsinki metropolitan area despite the shorter follow-up time. (Table 1) Moreover, the PTR was high at both times in 82% of the metropolitan area schools, when the corresponding percentage in other areas was 47%. The pupil socioeconomic composition was high at both times in 59% of the metropolitan area schools, when the corresponding percentage in other areas was 26%. (Data not shown in tables.)

We tested whether the school location/follow-up time acted as an effect modifier in the association between IAQ and teacher sick leave. The interaction effect (PTR*IAQ) was however nonsignificant (p > .60). Thus, school location/follow-up time was used as a covariate in further analyses.

At baseline (in 2001–02), 58% (n = 971) had no sick leaves, 25% (n = 416) had short-term sick leaves, and 17% (n = 291) had long-term sick leaves (with or without short-term sick leaves). Both short- and long-term sick leaves increased during the follow-up: at follow-up (in 2004–05), of the teachers in the study, 37% (n = 618) had no sick leaves; 35% (n = 581) had short-term sick leaves only; and 28% (n = 479) had long-term sick leaves (with or without short-term sick leaves) in 2004–05. In the empty model (Table 2), variance in teachers’ sick leaves between schools or municipalities was nonsignificant, i.e., no schools or municipalities had either a particularly large or particularly small number of teacher sick leaves.

**Table 2:** Summary of random effects of associations between changing school environment and teacher sick leave

	***School variance***	***SE***	***P value***	***Municipality variance***	***SE***	***P value***
**Short-term**						
Empty	0.056	0.043	0.10	0	-	-
with IAQ	0.019	0.037	0.20	0	-	-
Model I*	0.031	0.042	0.23	0.039	0.036	0.14
Model II**	<0.001	-	-	0.028	0.035	0.21
**Long-term**						
Empty	0.038	0.049	0.22	0.016	0.021	0.21
with IAQ	0.029	0.047	0.27	0.017	0.021	0.21
Model I*	<0.001	-	-	0.121	0.083	0.07
Model II**	<0.001	-	-	0.084	0.076	0.13

* Model adjusted for follow-up time, teachers’ sex, age, employment contract, occupation, and teacher sick leaves during 2001–2002. ** Model adjusted as Model I + pupil-teacher ratio, pupil cohort socioeconomic composition, school satisfaction, and bullying at school from baseline to follow-up.

Multinomial regression models.

### School environment as predictor of teacher sick leave

The pupil-reported IAQ improved slightly during the follow-up (t = 14.8[DF 1677], p < 0.001). The mean level of perceived IAQ was 2.7 (SD = 0.2) at baseline and 2.6 (SD = 0.2) at follow-up. At follow-up, 1072 (64%) teachers were in schools with above median perceived IAQ, and 606 (36%) were below the baseline determined median of perceived IAQ. Both the unadjusted and adjusted associations between the level of or change in perceived IAQ and teachers’ short-term sick leave are presented in the first part of Table 3, and the associations with long-term sick leaves are in the second part of Table 3 (Additional file 1: Table S1).

**Table 3:** Change in or the level of pupil-reported school environment as predictor of teachers’ short-term sick leave (vs. no sick leaves) in 2004–05

**Teacher characteristics**	**Unadjusted model**		Model I*		Model II**	
	**OR (95% CI)**	**P value**	**OR (95% CI)**	**P value**	**OR (95% CI)**	**P value**
Women vs. men			1.41 (1.08-1.86)	0.01	1.45 (1.10-1.91)	0.009
Age/10 years			0.66 (0.57-0.76)	<0.001	0.67 (0.58-0.77)	<0.001
Special vs. general education (2004–05)			0.82 (0.49-1.38)	0.46	0.90 (0.53-1.50)	0.68
Fixed-term vs. permanent job (2004–05)			0.88 (0.62-1.26)	0.49	0.89 (0.62-1.27)	0.52
Short-term sick leaves in 01–02: yes vs. no			3.05 (2.22-4.19)	<0.001	3.14 (2.28-4.32)	<0.001
**School characteristics**						
School location/follow-up time: 2 vs. 4 years			1.20 (0.79-1.83)	0.39	1.22 (0.78-1.91)	0.38
High vs. small PTR at school***					0.84 (0.54-1.29)	0.41
Low vs. high pupil socioeconomic composition****					0.63 (0.43-0.90)	0.01
**Pupil school satisfaction**						
1 Poor at both times, 22 schools (n = 414)					1.00 = Referent	
2 Good at both times, 36 schools (n = 619)					1.00 (0.69-1.44)	0.99
3 Negative change; from good to poor, 13 schools (n = 232)					1.78 (1.13-2.81)	0.01
4 Positive change; from poor to good, 22 schools (n = 413)					1.44 (0.98-2.11)	0.06
**Pupils being bullied**						
1 Poor at both times, 27 schools (n = 501)					Referent	
2 Good at both times, 29 schools (n = 488)					1.15 (0.79-1.69)	0.47
3 Negative change; from good to poor, 18 schools (n = 350)					0.97 (0.68-1.40)	0.89
4 Positive change; from poor to good, 19 schools (n = 339)					0.90 (0.60-1.36)	0.61
**Pupils bullying others**						
1 Poor at both times, 28 schools (n = 464)					Referent	
2 Good at both times, 28 schools (n = 549)					0.96 (0.66-1.40)	0.83
3 Negative change; from good to poor, 16 schools (n = 287)					1.51 (1.01-2.25)	0.04
4 Positive change; from poor to good, 21 schools (n = 378)					1.42 (0.97-2.08)	0.07
**Indoor air quality**						
1 Poor at both times, 28 schools (n = 485)	1.00 = Referent		Referent		Referent	
2 Good at both times, 37 schools (n = 699)	0.60 (0.45-0.79)	<0.001	0.63 (0.46-0.86)	0.003	0.61 (0.45-0.85)	0.003
3 Negative change; from good to poor, 7 schools (n = 121)	0.80 (0.50-1.30)	0.37	0.78 (0.50-1.30)	0.34	0.80 (0.47-1.35)	0.40
4 Positive change; from poor to good, 21 schools (n = 373)	0.59 (0.42-0.83)	0.002	0.64 (0.45-0.92)	0.02	0.60 (0.41-0.88)	0.009

* Model adjusted for teachers’ sex, age, employment contract, occupation, sick leaves during 2001–2002, and school location/follow-up time. ** Model adjusted as Model I + pupil-teacher ratio, pupil cohort socioeconomic composition, school satisfaction, and bullying at school from baseline to follow-up. *** Indicates above baseline median pupil-teacher ratio (>10.29) both at baseline and at follow-up vs. below baseline median or decreased pupil-teacher ratio. **** Indicates the percentage of pupils at school whose mothers had no more than a vocational education both at baseline and at follow-up and those with a negative change (low pupil cohort socioeconomic composition) vs. the percentage of pupils whose mothers have higher than a vocational education at both times high pupil cohort socioeconomic composition).

Multinomial logistic regression.

In the fully adjusted model, teachers in schools with good perceived IAQ at both times were at a 0.61 -fold (95% CI: 0.45-0.85) decreased risk of short-term sick leave compared to teachers in schools in which the perceived IAQ was poor at both times. The corresponding OR was 0.60 (95% CI: 0.41-0.88) when we compared these teachers to teachers in schools in which a beneficial change in perceived IAQ had occurred. A negative change in pupil school satisfaction (OR = 1.78, 95% CI: 1.13-2.81) and in pupils bullying others (OR = 1.51, 95% CI: 1.01-2.25) increased the risk of teachers’ short-term sick leaves compared to schools in which pupil satisfaction/pupil bullying was at poor, but stable, at both times.

The level of or change in pupil cohort socioeconomic composition was also associated with teacher short-term sick leaves. Teachers in schools with lower pupil socioeconomic composition at both times were at a *decreased* risk of short-term sick leaves compared to teachers in schools with higher pupil socioeconomic composition at both times (OR = 0.63, 95% CI: 0.43-0.90). Other factors predicting teacher short-term sick leaves were prior sick leaves, female gender, and younger age. School location/follow-up time was not associated with teacher sick leaves.

The level of or change in school environment factors were not associated with teachers’ long-term sick leaves. Factors associated with long-term sick leaves were: prior sick leaves, female gender, younger age, and permanent job contract. (Additional file 1: Table S1, continued from Table 3).

### The relationship of IAQ and pupil-related psychosocial factors in predicting teacher sick leave

We found no evidence of mediation of pupil-related psychosocial factors between IAQ and sick leaves, i.e., the odds ratios were similar in the unadjusted and adjusted models (Table 3). We also tested for whether there was an interaction (effect modification) of change in IAQ and change in school satisfaction/pupils bullying others in explaining teacher sick leaves. The interaction effects were however nonsignificant (p > .50). Neither was there an interaction of change in IAQ and change in PTR (p>. 30), or change in pupil socioeconomic composition (p > .90).

## Discussion

In this prospective study of 1678 teachers in 93 Finnish lower secondary schools, we examined whether school environment, as indicated by pupil-reported IAQ and pupil-related psychosocial factors predicted teacher sick leave. We found that good perceived IAQ at school was associated with decreased the risk of teachers’ short-term sick leave even after adjustment for baseline sick leaves, level of or a change in pupil cohort socioeconomic composition, the PTR at school, and pupil-related psychosocial factors. Moreover, improved IAQ at school also decreased the risk for short-term sick leaves.

The study is unique; we are not aware of any published studies regarding this issue. The results are plausible as short-term sick leaves are an indicator of minor or short-term health problems [31], and poor IAQ has been linked to minor health problems [3]. Along with poor perceived IAQ, negative changes in the pupil-related psychosocial factors seemed to increase the risk of teachers’ short-term sick leaves.

Based on this study, it seems that lower pupil cohort socioeconomic composition, pupil-related psychosocial problems, and prolonged problems in IAQ were somewhat clustered in the same schools, even though their effects on teacher sick leaves were different and independent from each other. Contrary to our expectations, there were more teachers’ short-term sick leaves in schools with higher pupil cohort socioeconomic composition than in schools with lower pupil cohort socioeconomic composition. This was however explained by the school location: short-term sick leaves were more prevalent in schools located in the metropolitan area, where the socioeconomic composition of pupils was higher than in other areas. Consequently, also IAQ problems were more prevalent in the metropolitan schools. However, the school location did not moderate the effect of IAQ on short-term leaves: when the IAQ was constantly poor, the risk for short-term leaves increased despite of school location.

Previous studies have suggested that the psychosocial work environment might contribute to the link between IAQ and health [3,18-23]. Comparison of models with and without adjustment for pupil-related psychosocial factors did not however indicate mediation or effect modification.

The major strengths of our study were that we were able to use exposure and outcome variables derived from independent data sources, objective measurement of sick leave from records with good coverage, and multilevel modeling taking into account the hierarchical nature of the data. We were also able to control for a large number of covariates on both the individual and school level, and use a prospective study design. As our measures came from independent sources, common method bias cannot explain our findings on the detrimental effects of constantly poor IAQ and pupil-related psychosocial problems on teacher health. Moreover, the survey on IAQ was conducted on the same month each year. Thus, the perceived changes in IAQ cannot be due to seasonal changes.

However, although the use of subjective evaluations of school IAQ is considered cost-effective, and these evaluations have shown associations with objective measures of IAQ [10-15], the subjective measurement of IAQ is also a limitation of the study. Pupil evaluations on whether poor ventilation or indoor air at school hinders one’s school work may reflect, in addition to actual IAQ, pupils’ awareness of air quality factors, susceptibility to air quality factors, or psychological or socioeconomic factors indicating propensity to attribute school work performance to external (environmental) factors. Moreover, based on these subjective evaluations, we cannot evaluate what is specifically wrong with indoor air or ventilation at these schools. The schools may be either too warm or too cold; the air may be either stuffy or drafty. Along with poorer perceived IAQ, feelings of thermal discomfort (thermally warm) have been associated with poorer performance and physiological symptoms [32], Previous research has also confirmed that of the indoor air pollutants, asbestos, carbon monoxide and carbon dioxide, nitrogen dioxide, formaldehyde, radon, VOCs, indoor biological allergens, fungi, bacteria, and viruses are associated with ill-health and poorer functioning [3]. Moreover, outdoor pollutants may also contribute to indoor air quality [33].

Another limitation of this study may relate to the healthy worker effect [34]. It is possible that those that remained in the study group during the follow-up were healthier than those that dropped out, i.e., those who were no longer employed at follow-up, and thus were excluded from our analyses. This would suggest that the effects of IAQ on health would be underestimates.

Since lower secondary school in Finland lasts for three years (grades 7-9), the pupils evaluating IAQ and the psychosocial school environment in 2001/02, and those evaluating them in 2004/05 were largely different. This can be considered either a strength or a weakness; evaluations from different pupils decrease common method bias, but may also mean that the changes were not necessarily in the variables investigated but in pupil composition. Pupil composition, with regard to follow-up time and the level of or change in pupil cohort socioeconomic composition was, however, controlled for in our analysis.

## Conclusion

To conclude, problems in the school environment, as indicated by poor IAQ, as well as pupil school dissatisfaction and bullying, are associated with increased risk of short-term sick leaves among teachers. The fact that sick leave levels decreased among teachers from schools in which perceptions of IAQ improved, suggests that such improvements have potentially positive effects on the health and well-being of teachers and on the costs incurred by health care and absenteeism, as well as on the well-being of pupils. Finally, constantly poor IAQ, psychosocial problems and low pupil socioeconomic composition seem to cluster in certain schools often located in metropolises.

## Competing interests

The authors declare that they have no competing interests.

## Authors’ contributions

JE was the principal author of the paper and carried out the data analyses with help from JP and MV, who supervised the data analyses and participated in designing the study, and writing the paper. MK ja JV are the directors of the Finnish Public Sector Study; they helped in writing the paper and interpreting the results. RP is the director of the School Health Promotion Study, and participated in interpreting the results. TP, IK, SVS, and TO contributed to the interpretation of the results and edited the manuscript. All authors read and approved the final manuscript.

## Pre-publication history

The pre-publication history for this paper can be accessed here:

http://www.biomedcentral.com/1471-2458/12/770/prepub

## Supplementary Material

Additional file 1 ** Change in or the level of pupil-reported school environment as predictor of teachers’ long-term sick leave (with or without short-term sick leaves) vs. no sick leaves in 2004–05.** Multinomial logistic regression. Click here for file

## References

[B1] United States Environmental Protection Agency: IAQ tools for schools. Health and achievement. Common symptoms http://www.epa.gov/iaq/schools/symptoms.html

[B2] Reijula K Sundman-Digert C Assessment of indoor air problems at work with a questionnaire Occup Environ Med 2004 61 33 38 14691270 PMC1757823

[B3] Jones AP Indoor air quality and health Atmos Environ 1999 33 4535 4564 10.1016/S1352-2310(99)00272-1

[B4] Mendell MJ Fisk WJ Kreiss K Levin H Alexander D Cain WS Girman JR Hines CJ Jensen PA Milton DK Rexroat LP Wallingford KM Improving the health of workers in indoor environments: priority research needs for a national occupational research agenda Am J Public Health 2002 92 1340 1440 10.2105/ajph.92.9.1430 PMC1447254 12197969

[B5] Kolarik J Wargocki P Can a photocatalytic air purifier be used to improve the perceived air quality indoors? Indoor Air 2010 20 255 262 10.1111/j.1600-0668.2010.00650.x 20573125

[B6] Moglia D Smith A MacIntosh DL Somers JL Prevalence and implementation of IAQ programs in U.S. schools Environ Health Persp 2006 114 141 146 10.1289/ehp.7881 PMC1332670 16393672

[B7] Daisey JM Angell WJ Apte MG Indoor air quality, ventilation and health symptoms in schools: an analysis of existing information Indoor Air 2003 13 53 64 10.1034/j.1600-0668.2003.00153.x 12608926

[B8] Haverinen-Shaughnessy U Moschandreas DJ Shaughnessy RJ Association between substandard classroom ventilation rates and students’ academic achievement Indoor Air 2011 21 121 131 10.1111/j.1600-0668.2010.00686.x 21029182

[B9] Mendell MJ Heath GA Do indoor pollutants and thermal conditions in schools influence student performance? A critical review of the literature Indoor Air 2005 15 27 52 15660567 10.1111/j.1600-0668.2004.00320.x

[B10] Fang L Wyon DP Clausen G Fanger PO Impact of indoor air temperature and humidity in an office on perceived air quality, SBS symptoms and performance Indoor Air 2004 14 74 81 15330775 10.1111/j.1600-0668.2004.00276.x

[B11] Wargocki P Wyon DP Baik YK Clausen G Fanger PO Perceived air quality, sick building syndrome (SBS) symptoms and productivity in an office with two different pollution loads Indoor Air 1999 9 165 179 10.1111/j.1600-0668.1999.t01-1-00003.x 10439554

[B12] Engvall K Wickman P Norbäck D Sick building syndrome and perceived indoor environment in relation to energy saving by reduced ventilation flow during heating season: a 1 year intervention study in dwellings Indoor Air 2005 15 120 126 10.1111/j.1600-0668.2004.00325.x 15737154

[B13] Hellgren U-M Hyvärinen M Holopainen R Reijula K Perceived indoor air quality, air-related symptoms and ventilation in Finnish hospitals Int J Occup Med Environ Health 2011 24 48 56 10.2478/s13382-011-0011-5 21468902

[B14] Seppänen OA Fisk WJ Summary of human responses to ventilation Indoor Air 2004 14 Suppl 7 102 118 15330778 10.1111/j.1600-0668.2004.00279.x

[B15] Claeson A-S Nordin S Sunesson A-L Effects on perceived air quality and symptoms of exposure to microbially produced metabolites and compounds emitted from damp building materials Indoor Air 2009 19 102 112 10.1111/j.1600-0668.2008.00566.x 19077173

[B16] Brauer C Budtz-Jørgesen E Mikkelsen S Structural equation analysis of the causal relationship between health and perceived indoor environment Int Arch Occup Environ Health 2008 81 769 776 10.1007/s00420-007-0244-6 17917740

[B17] Hemingway H Marmot M Psychosocial factors in the aetiology and prognosis of coronary heart disease: systematic review of prospective cohort studies BMJ 1999 318 1460 1467 10.1136/bmj.318.7196.1460 10346775 PMC1115843

[B18] Hansen ÅM Meyer HW Gyntelberg F Building-related symptoms and stress indicators Indoor Air 2008 18 440 446 10.1111/j.1600-0668.2008.00571.x 18823341

[B19] Bakke JV Moen BE Wieslander G Norbäck D Gender and the physical and psychosocial work environments are related to indoor air symptoms J Occup Environ Med 2007 49 641 650 10.1097/JOM.0b013e31806e5fa0 17563607

[B20] Lahtinen M Huuhtanen P Reijula K Sick building syndrome and psychosocial factors—a literature review Indoor Air 1998 4 71 80

[B21] Lahtinen M Huuhtanen P Kähkönen E Reijula K Psychosocial dimensions of solving an indoor air problem Indoor Air 2002 12 33 46 10.1034/j.1600-0668.2002.120105.x 11951709

[B22] Meyer HW Würtz H Suadicani P Valbjørn O Sigsgaard T Gyntelberg F The DAMIB group Molds in floor dust and building-related symptoms in adolescent school children Indoor Air 2004 14 65 72 14756847 10.1046/j.1600-0668.2003.00213.x

[B23] Lahtinen M Sundman-Digert C Reijula K Psychosocial work environment and indoor air problems: a questionnaire as a means of problem diagnosis Occup Environ Med 2004 61 143 149 10.1136/oem.2002.005835 14739380 PMC1740705

[B24] United States Environmental Protection Agency: What factors in schools contribute to poor IAQ? http://iaq.supportportal.com/ics/support/kbAnswer.asp?deptID=23007&task=knowledge&questionID=21854

[B25] United States Environmental Protection Agency: How big a problem is poor indoor air quality in schools? http://iaq.supportportal.com/ics/support/kbAnswer.asp?deptID=23007&task=knowledge&questionID=16120

[B26] Simons E Hwang S-A Fitzgerald EF Kielb C Lin S The impact of school building conditions on student absenteeism in upstate New York Am J Public Health 2010 100 1679 1686 10.2105/AJPH.2009.165324 20634471 PMC2920982

[B27] Ervasti J Kivimäki M Puusniekka R Luopa P Pentti J Suominen S Ahola K Vahtera J Virtanen M Students’ school satisfaction as predictor of teacher sick leave: a prospective cohort study Eur J Public Health 2012 22 215 219 10.1093/eurpub/ckr043 21504980

[B28] Ervasti J Kivimäki M Puusniekka R Luopa P Pentti J Suominen S Vahtera J Virtanen M Association of pupil vandalism, bullying and truancy with teachers’ absence due to illness: a multilevel analysis J Sch Psychol 2012 50 347 361 10.1016/j.jsp.2011.11.006 22656077

[B29] Virtanen M Kivimäki M Pentti J Oksanen T Ahola K Linna A Kouvonen A Salo P Vahtera J School neighbourhood disadvantage as a predictor of long-term sick leave among teachers: prospective cohort study Am J Epidemiol 2010 171 785 792 10.1093/aje/kwp459 20179159

[B30] Ervasti J Kivimäki M Kawachi I Subramanian SV Pentti J Ahola K Oksanen T Pohjonen T Vahtera J Virtanen M Pupils with special educational needs in basic education schools and teachers’ sickness absences - a register-linkage study Scand J Work Environ Health 2012 38 209 217 22344461 10.5271/sjweh.3281

[B31] Marmot M Feeney A Shipley M North F Syme SL Sickness absence as a measure of health status and functioning: from the UK Whitehall II study J Epidemiol Community Health 1995 49 124 130 10.1136/jech.49.2.124 7798038 PMC1060095

[B32] Lan L Wargocki P Wyon DP Lian Z Effects of thermal discomfort in an office on perceived air quality, SBS symptoms, physiological responses, and human performance Indoor Air 2011 21 376 390 10.1111/j.1600-0668.2011.00714.x 21306437

[B33] Graveland H Van Roosbroeck SAH Rensen WM Brunekreef B Gehring U Air pollution and exhaled nitric oxide in Dutch schoolchildren Occup Environ Med 2011 68 551 556 10.1136/oem.2010.056812 21045215

[B34] Carpenter LM Some observations on the healthy worker effect Br J Ind Med 1987 44 289 291 3593659 10.1136/oem.44.5.289 PMC1007826

