# Supplementary material for: School environment as predictor of teacher sick leave: data-linked prospective cohort study
Source: BMC Public Health. 2012 Sep 11;12:770. doi: 10.1186/1471-2458-12-770 (PMC3490775; doi:10.1186/1471-2458-12-770)
Supplement: Additional file 1 — Change in or the level of pupil-reported school environment as predictor of teachers’ long-term sick leave (with or without short-term sick leaves) vs. no sick leaves in 2004–05. Multinomial logistic regression. [file 1471-2458-12-770-S1.doc]

Additional file 1 (continued from Table 3). Change in or the level of pupil-reported school environment as predictor of teachers' long-term sick leave (with or without short-term sick leaves) vs. no sick leaves in 2004-05. Multinomial logistic regression.

| **Teacher characteristics** | **Unadjusted model** |  | **Model I*** |  | **Model II**** |  |
| --- | --- | --- | --- | --- | --- | --- |
|  | **OR (95% CI)** | **P value** | **OR (95% CI)** | **P value** | **OR (95% CI)** | **P value** |
| **Women vs. men** |  |  | 1.46 (1.08-1.95) | 0.01 | 1.48 (1.10-1.99) | 0.01 |
| Age / 10 years |  |  | 0.74 (0.63-0.86) | <0.001 | 0.75 (0.64-0.88) | <0.001 |
| Special vs. general education |  |  | 0.90 (0.53-1.55) | 0.71 | 0.93 (0.54-1.60) | 0.80 |
| Fixed-term vs. permanent job |  |  | 0.65 (0.44-0.98) | 0.04 | 0.64 (0.42-0.95) | 0.03 |
| Long-term sick leaves in 01-02: yes vs. no |  |  | 4.06 (2.78-5.93) | <0.001 | 4.20 (2.86-6.17) | <0.001 |
| School characteristics |  |  |  |  |  |  |
| School location/follow-up time: 2 vs. 4 years |  |  | 1.28 (0.70-2.31) | 0.42 | 1.50 (0.83-2.69) | 0.18 |
| High vs. small PTR at school*** |  |  |  |  | 0.68 (0.43-1.08) | 0.10 |
| Low vs. high pupil socioeconomic composition**** |  |  |  |  | 0.78 (0.53-1.16) | 0.22 |
| **School satisfaction** |  |  |  |  |  |  |
| 1 Poor at both times, 22 schools (n=414) |  |  |  |  | 1.00=Referent |  |
| 2 Good at both times, 36 schools (n=619) |  |  |  |  | 0.98 (0.67-1.45) | 0.94 |
| 3 Negative change; from good to poor, 13 schools (n=232) |  |  |  |  | 1.56 (0.96-2.54) | 0.07 |
| 4 Positive change; from poor to good, 22 schools (n=413) |  |  |  |  | 1.04 (0.69-1.58) | 0.84 |
| **Pupils being bullied** |  |  |  |  |  |  |
| 1 Poor at both times, 27 schools (n=501) |  |  |  |  | Referent |  |
| 2 Good at both times, 29 schools (n=488) |  |  |  |  | 1.11 (0.74-1.67) | 0.62 |
| 3 Negative change; from good to poor, 18 schools (n=350) |  |  |  |  | 0.71 (0.47-1.05) | 0.09 |
| 4 Positive change; from poor to good, 19 schools (n=339) |  |  |  |  | 0.81 (0.53-1.26) | 0.35 |
| **Pupils bullying others** |  |  |  |  |  |  |
| 1 Poor at both times, 28 schools (n=464) |  |  |  |  | Referent |  |
| 2 Good at both times, 28 schools (n=549) |  |  |  |  | 0.99 (0.65-1.49) | 0.95 |
| 3 Negative change; from good to poor, 16 schools (n=287) |  |  |  |  | 1.41 (0.92-2.15) | 0.11 |
| 4 Positive change; from poor to good, 21 schools (n=378) |  |  |  |  | 1.27 (0.84-1.89) | 0.25 |
| **Indoor air quality** |  |  |  |  |  |  |
| 1 Poor at both times, 28 schools (n=485) | 1.00=Referent |  | Referent |  | Referent |  |
| 2 Good at both times, 37 schools (n=699) | 0.74 (0.54-1.02) | 0.06 | 0.78 (0.57-1.07) | 0.13 | 0.76 (0.54-1.07) | 0.12 |
| 3 Negative change; from good to poor , 7 schools (n=121) | 0.76 (0.44-1.31) | 0.33 | 0.80 (0.47-1.38) | 0.43 | 0.82 (0.46-1.46) | 0.49 |
| 4 Positive change; from poor to good, 21 schools (n=373) | 0.94 (0.66-1.33) | 0.72 | 1.03 (0.72-1.48) | 0.87 | 0.94 (0.63-1.41) | 0.78 |

* Model adjusted for teachers' sex, age, employment contract, occupation, and teacher sick leaves during 2001-2002, and school location/follow-up time. ** Model adjusted as Model I+ pupil-teacher ratio, pupil cohort socioeconomic composition, school satisfaction, and bullying at school from baseline to follow-up *** Indicates above baseline median pupil-teacher ratio (>10.29) both at baseline and at follow-up vs. below baseline median or decreased pupil-teacher ratio. **** Indicates the percentage of pupils at school whose mothers had no more than a vocational education both at baseline and at follow-up and those with a negative change (low pupil cohort socioeconomic composition) vs. the percentage of pupils whose mothers have higher than a vocational education at both times high pupil cohort socioeconomic composition).
